# Consideration of clinicopathologic features improves patient stratification for multimodal treatment of gastric cancer

**DOI:** 10.18632/oncotarget.18607

**Published:** 2017-06-22

**Authors:** In Cho, In Gyu Kwon, Ali Guner, Taeil Son, Hyoung-Il Kim, Dae Ryong Kang, Sung Hoon Noh, Joon Seok Lim, Woo Jin Hyung

**Affiliations:** ^1^ Department of Surgery, Graduate School, Yonsei University College of Medicine, Seoul, Republic of Korea; ^2^ Department of Surgery, Soonchunhyang University College of Medicine, Bucheon, Republic of Korea; ^3^ Department of Surgery, Keimyung University School of Medicine, Daegu, Republic of Korea; ^4^ Department of General Surgery, Karadeniz Technical University, Trabzon, Turkey; ^5^ Department of Surgery, Yonsei University College of Medicine, Seoul, Republic of Korea; ^6^ Gastric Cancer Center, Yonsei Cancer Center, Yonsei University Health System, Seoul, Republic of Korea; ^7^ Department of Humanities and Social Medicine, Ajou University School of Medicine, Suwon, Republic of Korea; ^8^ Department of Diagnostic Radiology, Yonsei University College of Medicine, Seoul, Republic of Korea; ^9^ Robot and MIS Center, Severance Hospital, Yonsei University Health System, Seoul, Republic of Korea

**Keywords:** gastric cancer, preoperative staging, diagnostic accuracy, patient stratification, multimodal treatment

## Abstract

Preoperative staging of gastric cancer with computed tomography alone exhibits poor diagnostic accuracy, which may lead to improper treatment decisions. We developed novel patient stratification criteria to select appropriate treatments for gastric cancer patients based on preoperative staging and clinicopathologic features. A total of 5352 consecutive patients who underwent gastrectomy for gastric cancer were evaluated. Preoperative stages were determined according to depth of invasion and nodal involvement on computed tomography. Logistic regression analysis was used to identify clinicopathological factors associated with the likelihood of proper patient stratification. The diagnostic accuracies of computed tomography scans for depth of invasion and nodal involvement were 67.1% and 74.1%, respectively. Among clinicopathologic factors, differentiated tumor histology, tumors smaller than 5 cm, and gross appearance of early gastric cancer on endoscopy were shown to be related to a more advanced stage of disease on preoperative computed tomography imaging than actual pathological stage. Additional consideration of undifferentiated histology, tumors larger than 5 cm, and grossly advanced gastric cancer on endoscopy increased the probability of selecting appropriate treatment from 75.5% to 94.4%. The addition of histology, tumor size, and endoscopic findings to preoperative staging improves patient stratification for more appropriate treatment of gastric cancer.

## INTRODUCTION

A global health problem, gastric cancer ranks as the third most common cause of cancer-related deaths worldwide [1]. Despite efforts to improve early detection of gastric cancer by mass screening in Korea and Japan, the proportion of advanced gastric cancers still remains high at 39.2%-51.4% [2-4]. Indeed, the incidence of advanced gastric cancer is much higher in Western countries [2, 5]. Consequently, developing treatment strategies for patients with advanced gastric cancer has emerged as a major concern, and multimodal treatments, such as neoadjuvant chemotherapy and perioperative or preoperative chemoradiotherapy, have been applied for the treatment of advanced gastric cancer.

Major guidelines for gastric cancer treatment recommend that decisions are to be made based on clinical staging, primarily achieved with the use of computed tomography (CT) [6-8]. With advances in equipment, development of contrast materials, and applications of stomach cancer-specific protocols, the diagnostic accuracy of CT scans for gastric cancer has improved considerably. The overall diagnostic accuracies of CT scans in predicting tumor depth and nodal status range from 71.4% to 88.9% and 75.9% to 78%, respectively [9-12]. Nevertheless, such accuracies are insufficient for proper treatment decisions. Moreover, studies on preoperative, multimodal treatments have included patients with pathologically early stage gastric cancer who do not require any perioperative or preoperative chemotherapy.

Although many studies have evaluated the accuracy of clinical staging using CT scans for gastric cancer, most have analyzed its accuracy in regards to depth of invasion or nodal status separately. Few studies have focused on evaluating diagnostic accuracy in relation to overall clinical staging. Furthermore, no study has attempted to identify risk factors for inappropriate treatment decisions caused by inaccurate clinical staging, and no study has evaluated clinicopathologic features that may help improve patient stratification for proper decision making.

In the present study, we hypothesized that additional consideration of clinicopathologic features in clinical staging would facilitate proper patient stratification for gastric cancer treatment. Accordingly, we compared preoperative clinical stages to their actual pathologic stages to investigate the diagnostic accuracy of clinical staging with CT alone in gastric cancer and then evaluated which clinicopathologic features improved the diagnostic accuracy thereof. Additionally, we attempted to develop novel patient stratification criteria to select appropriate treatment modalities for gastric cancer patients based on preoperative staging and clinicopathologic factors found to be associated with improved diagnostic accuracy.

## RESULTS

### Patient characteristics

Patient demographics and clinicopathological characteristics are summarized in Table 1. There were 3491 men and 1861 women in the current study. The mean age was 57.9 ± 11.9 years. The mean tumor size was 3.6 ± 2.7cm. The tumors were located mainly in the lower third (58.8%) and lesser curvature (43.5%) of the stomach. Histologically, undifferentiated tumors were predominant (56.6%), compared to differentiated ones (43.4%). In preoperative clinical staging based on analysis of CT images, the majority of patients were classified as clinical T1 stage (52.3%) and clinical N- stage (69.4%). On pathological staging, T1 (56.3%) and N-(64.5%) tumors were most common.

**Table 1:** Demographics and clinicopathological features.

Variable	*N* = 5352	%
Age (years)	57.9 ± 11.9	
Gender		
Male	3491	65.2
Female	1861	34.8
Body mass index (kg/m2)	23.3 ± 2.9	
Tumor size (cm)	3.64 ± 2.65	
Gross finding		
EGC	3046	56.9
AGC	2306	43.1
Ulceration		
Absent	1910	35.7
Present	3442	64.3
Histology		
Differentiated	2324	43.4
Undifferentiated	3028	56.6
Location, circular		
Lesser curvature	2329	43.5
Greater curvature	735	13.7
Anterior wall	1061	19.8
Posterior wall	1178	22.0
Encircling	49	0.9
Location, tubular		
Upper	682	12.7
Middle	1514	28.3
Lower	3147	58.8
Entire	9	0.2

EGC, early gastric cancer; AGC, advanced gastric cancer

### Accuracy of preoperative staging

A comparison of T classification between the preoperative CT scans and final pathologic results is shown in Table 2. The accuracy of T classification was 67.1%, with overestimation occurring in 18.5% of patients and underestimation occurring in 14.4% of patients. The reliability for tumor depth was *k* = 0.65. The cT2-3 classification subgroup showed the highest percentage of overestimation (35.5%, 506/1424). The accuracy of preoperative N classification was 74.1% (3968/5352). Overestimation and underestimation of N classification reached 10.5% (563/5352) and 15.4% (821/5352), respectively. The sensitivity and specificity for clinical node involvement (cN+) were 65.6% and 83.7%, respectively. A comparison between preoperative and postoperative staging is shown in Table 3. The accuracy of overall staging was 53.3%; overestimation and underestimation reached 23.9% and 22.8%, respectively. The reliability for overall staging was *k* = 0.69.

**Table 2:** Correlation of clinical and pathologic stage for depth of invasion and nodal status.

Depth of invasion	pT1 ( *N* =3013)		pT2/T3 (*N* = 1244)		pT4 (*N* = 1095)		Total (*N* = 5352)
cT1	2392		319		86		2797
cT2–3	506		554		364		1424
cT4	115		371		645		1131
*k* value							0.65

Nodal involvement		pN(-) (*N* = 3454)		pN(+) (*N* = 1898)		Total (*N* = 5352)	
cN(-)		2891		821		3712	
cN(+)		563		1077		1640	
Sensitivity						65.6 %	
Specificity						83.7 %	

pT, pathological T staging; cT, clinical T staging; pN, pathological N staging; cN, clinical N staging; pT/N, overall pathological staging; cT/N, overall clinical staging.

**Table 3:** Correlation of overall clinical and pathologic stage.

Stage grouping	pT1/N(-) (*N* = 2700)	pT1/N(+) (*N* = 313)	pT2–3/N(-) (*N* = 565)	pT2–3/N(+) (*N* = 679)	pT4/N(-) (*N* = 189)	pT4/N(+) (*N* = 906)	Total (*N* = 5352)
cT1/N(-)	2017	166	168	86	26	38	2501
cT1/N(+)	175	34	31	34	5	17	296
cT2–3/N(-)	338	54	192	148	46	131	909
cT2–3/N(+)	78	36	67	147	36	151	515
cT4/N(-)	36	6	39	56	29	136	302
cT4/N(+)	56	17	68	208	47	433	829
*k* value							0.69

pT, pathological T staging; cT, clinical T staging; pN, pathological N staging; cN, clinical N staging; pT/N, overall pathological staging; cT/N, overall clinical staging.

In multivariate analysis of the cApE and cApA groups, undifferentiated histology (*p* < .001), tumors larger than 5 cm (*p* <.001), and grossly advanced gastric cancer (AGC) appearance on endoscopy (*p* <.001) were independently associated with a higher probability of proper patient stratification for treatment (cApA group) (Table 4).

**Table 4:** Clinicopathological features related to probability of proper patient stratification.

	A*	B^†^	Univariate analysis		Multivariate analysis		
	(*N* = 403)	(*N* = 1243)	OR (95% CI)	*P*		OR (95% CI)	*P*
Gender Male Female	285 118	854 389	Ref. 0.91 (0.71–1.16)	0.447		Ref. 0.92 (0.67–1.27)	0.619
Age (years)	403	1243	1.00 (0.99–1.01)	0.462		1.00 (0.99–1.01)	0.929
BMI (kg/m^2^)	403	1243	1.07 (1.03–1.11)	<0.001		1.05 (1.01–1.10)	0.029
Histology Differentiated Undifferentiated	207 196	396 847	Ref. 0.44 (0.35–0.56)	<0.001		Ref. 0.54 (0.40–0.73)	<0.001
Tumor size (cm) ≤5 >5	315 88	505 738	Ref. 0.19 (0.15–0.25)	<0.001		Ref. 0.31 (0.23–0.42)	<0.001
Gross findings EGC AGC	192 211	39 1204	Ref. 0.03 (0.02–0.05)	<0.001		Ref. 0.04 (0.03–0.07)	<0.001
Ulcerative No Yes	113 290	242 1001	Ref. 0.62 (0.48–0.80)	<0.001		Ref. 1.124 (0.76–1.67)	0.559
Location, circular Lesser curvature Greater curvature Anterior wall Posterior wall Encircling	203 49 68 81 2	672 176 168 192 35	Ref. 0.92 (0.65–1.31) 1.34 (0.97–1.85) 1.40 (1.03–1.89) 0.19 (0.05–0.79)	0.651 0.075 0.031 0.023		Ref. 0.74 (0.46–1.17) 1.23 (0.82–1.83) 1.14 (0.77–1.68) 0.36 (0.07–1.73)	0.198 0.315 0.525 0.202
Location, tubular Upper Middle Lower Entire	66 102 235 0	197 332 707 7	Ref. 0.92 (0.64–1.31) 0.99 (0.72–1.36) NA	0.634 0.961 NA		Ref. 0.78 (0.50–1.22) 0.75 (0.51–1.12) NA	0.280 0.156 NA

OR, odds ratio; EGC, early gastric cancer; AGC, advanced gastric cancer.

A*: Clinical early advancedwith pathological advanced early group.

B^†^: Clinical advanced with pathological advanced group.

### Probability for proper patient stratification

The probability of proper patient stratification was 75.5%, calculated as the number of patients in the cApA group (*n* = 1243) divided by the number in both the cApE and cApA groups (*n* = 1646) (Table 5). When we additionally considered the clinicopathological features found to be related to diagnostic accuracy in our study, the probability of proper patient stratification greatly improved. By considering only one additional feature (histology, size, or gross appearance), the probability of proper patient stratification improved from 75.5% to 81.2%-89.3%. Moreover, if two variables were considered, the probability increased to 88.0%-92.5%. Among patients with undifferentiated histology, tumors larger than 5 cm, and gross AGC appearance on endoscopy, the probability of proper patient stratification improved to 94.4%: in other words, candidates receiving potentially unnecessary neoadjuvant chemotherapy could be decreased from 403 patients (403/5352) to 30 patients (30/5352). Meanwhile, the probability of proper patient stratification was only 10.5% in patients who had differentiated histology, tumors smaller than 5 cm, and early gastric cancer (EGC) appearance on endoscopy.

**Table 5:** Probability of proper patient stratification when considering clinicopathological features.

(N)	Clinical advanced and pathological early group	Clinical advanced and pathological advanced group	Probability (%)
Variable not considered	403	1243	75.5
One variable considered			
AGC^§^	211	1204	85.1
> 5 cm*	88	738	89.3
Undiff^†^	196	847	81.2
Two variables considered			
AGC^§^ + > 5 cm*	59	724	92.5
> 5 cm* + Undiff^†^	46	520	91.9
Undiff^†^ + AGC^§^	111	820	88.0
Three variables considered			
AGC^§^ + > 5 cm* + Undiff^†^	30	507	94.4

AGC§: grossly advanced gastric cancer appearance in endoscopy; > 5 cm*: tumor size larger than 5 cm; Undiff†: undifferentiated histology.

## DISCUSSION

In the present study, the accuracies of estimating depth of invasion and nodal involvement on CT scans alone were 67.1% and 74.1%, respectively. The accuracy of overall staging was only 53.3%. More specifically, 35.5% and 34.3% of patients were overestimated for clinical T2-3 classification and node positivity, respectively. When we additionally considered the clinicopathologic features identified in our study to be associated with proper patient stratification, the probability thereof improved from 75.5% to 94.4%.

When deciding on a treatment modality before surgery, physicians must take into account the integrated clinical stage of each patient; however, preoperative staging based on CT scans alone can be inaccurate. Nevertheless, few studies have attempted to evaluate the accuracy of overall clinical staging using CT scans alone. In the current study, we found that the accuracy thereof in gastric cancer patients was only 53.3%.

Treatment modalities are commonly selected according to clinical stage. Japanese gastric cancer treatment guidelines state that there is no established benefit of neoadjuvant chemotherapy for gastric cancer patients. Meanwhile, Western guidelines recommend neoadjuvant chemotherapy or surgery for patients with stage II disease or higher (cT2-3N+ or cT4N-/+) [13]. The present study, however, found that the probability of delivering inappropriate treatment was 24.5% when relying on CT scans alone to conduct preoperative clinical staging. When we considered clinicopathologic features of histology, tumor size, and endoscopic findings, the probability of delivering an inappropriate treatment modality decreased to 5.6%.

Our results indicate that undifferentiated histology, tumors larger than 5 cm, and grossly AGC appearance on endoscopy are independently associated with a higher probability of reaching a proper treatment decision. To the best of our knowledge, this study is the first to evaluate clinicopathologic features that can improve stratification of patients with gastric cancer. When we applied the identified clinicopathologic features, the likelihood of proper patient stratification increased from 75.5% to 94.4%. Therefore, by considering these clinicopathologic features, physicians can spare patients with early staged tumors, who can be cured by surgery alone, from unnecessary pretreatment.

Neoadjuvant chemotherapy has been commonly used to treat resectable gastric cancer in Western countries. Its application is based on two large phase III clinical trials [13, 14], which demonstrated that perioperative chemotherapy facilitated significant improvements in resectability, disease-free survival, and overall survival, compared to surgery alone. However, despite these favorable results, the studies were critically limited in that no standard clinical staging method was applied at the time of randomization. As a result, patients in the surgery-only group with pT1 and pN- classification comprised 8.3% and 26.9%, respectively, of patients in the MAGIC trial [13]. Also, in the FNCLCC/FFCD trial [14], patients with a pN- classification accounted for 20% of the surgery-only group [13, 14]. This means that similar proportions of patients that did not need chemotherapy were included in the perioperative chemotherapy groups in those studies. Accordingly, physicians should caution against applying unnecessary neoadjuvant chemotherapy to early stage patients, as doing so introduces greater medical expense, causes suffering due to serious side effects, and postpones the timing of surgery. By applying the clinicopathologic risk factors shown to be significant for proper patient satisfaction in the current study, which are readily collected during routine patient evaluations, physicians can protect their patients from unnecessary neoadjuvant chemotherapy. In our study, the number of candidates receiving unnecessary neoadjuvant chemotherapy could have been decreased from 403 to 30 patients among 5352 patients.

This study is superior to previous studies for the following reasons: First, we utilized data from a large-scale database collected over a relatively short period. Additionally, CT scans and resected specimens were evaluated, respectively, by radiologists and pathologists specializing in upper gastroenterology at a single, high-volume center. As well, preoperative staging was evaluated using CT scans acquired following a stomach cancer-specific protocol. Therefore, the data were of similar quality and highly reliable.

Notwithstanding, there are several limitations to the current study that warrant consideration. First, there may be bias in our study due to the retrospective nature of the analyses, although we used a large-scale, homogenous database. First, there may be bias in our study due to the retrospective nature of the analyses, although we used a large-scale, homogenous database. Additionally, our use of two different gastric distension methods could have affected the evaluation of clinical T classification, and the possibility of inter-radiologist differences in evaluation of CT images could not be excluded, although we used the same criteria for clinical T and N classification. Therefore, bias in deciding clinical T and N factors could not be completely avoided. Moreover, since we excluded patients who received neoadjuvant chemotherapy, bias related to this exclusion might be present in the analysis cApE and cApA patients. Second, we did not consider other diagnostic modalities, such as diagnostic laparoscopy and endoscopic ultrasound. If additional diagnostic tools were included for preoperative staging, diagnostic accuracy may have been increased. Nevertheless, CT scans are non-invasive and are currently regarded as a standard staging method. It is, therefore, worth noting that a simple combination of CT scans and clinicopathologic features improves patient stratification. Third, the preoperative nodal staging system utilized in this study differed from the AJCC pathologic staging system. Although we recognized differences between the two systems, we were unable to take them into account due to the retrospective design of our study. Further prospective studies with various diagnostic modalities are necessary to validate our findings. Fourth, using the three factors we have identified, underdiagnosis would likely be increased. However, surgery with adjuvant chemotherapy would not be considered undertreatment, rather it is a standard treatment option for stage II or III gastric cancer patients. Since the purpose of this study was to develop novel stratification criteria with which to select proper gastric cancer patients for neoadjuvant chemotherapy, we have focused on clinically advanced cancers. Lastly, due to the retrospective nature of the study, we discerned histology from pathologic evaluation of resected specimens. Thus, the possibility of changes in histologic type after resection must be considered, although discrepancies in histologic diagnosis between biopsy and pathologies of resected specimens reportedly range only from 2.4% to 4.4% [15-17].

Current practices for treating gastric cancer based on preoperative staging with CT scans alone are associated with a high probability of improper treatment decisions due to low diagnostic accuracy. By additionally considering clinicopathologic features of histology, tumor size, and endoscopic findings, more appropriate treatment strategies can be established.

## MATERIALS AND METHODS

### Patients and clinicopathological features

**Figure 1:**
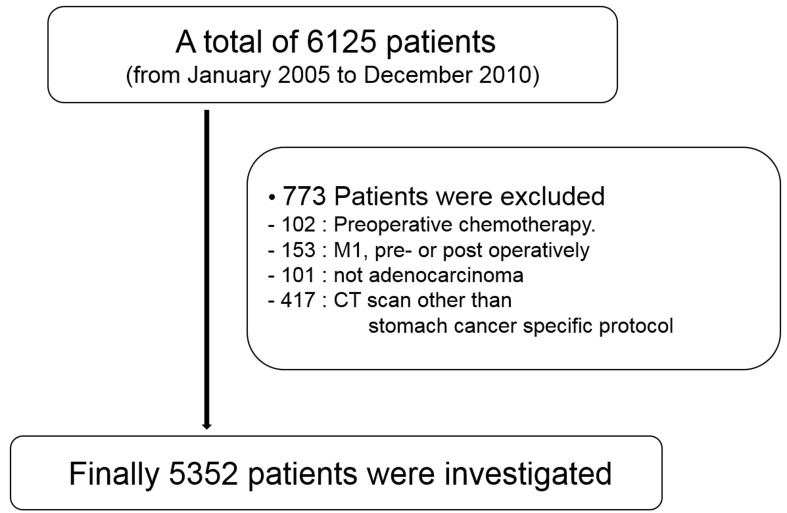
Flow diagram of study patients

A retrospective review of a prospectively maintained database for gastric cancer revealed a total of 6125 consecutive patients who underwent gastrectomy for primary gastric cancer from January 2005 to December 2010. We excluded patients from the analysis who fulfilled the following criteria: (1) received neoadjuvant therapy (*n* = 102); (2) received a preoperative or postoperative diagnosis of distant metastasis (*n* = 153); (3) did nothave adenocarcinoma (*n* = 101); and (4) were not examined with CT following a stomach cancer-specific protocol (*n* = 417). Finally, 5352 patients were included for analysis. (Figure 1) This study was reviewed and approved by the Institutional Review Board of Severance Hospital, Yonsei University Health System (4-2013-0510).

The demographics of the patients, including age, sex, and pathological features of histology, size, location, presence of ulceration, and gross type on endoscopy, were evaluated. Histologic types were classified into two groups: differentiated, which included papillary, well-differentiated, or moderately-differentiated adenocarcinoma, and undifferentiated, which included poorly-differentiated or undifferentiated adenocarcinoma, signet ring cell carcinoma, and mucinous carcinoma. Gross types were classified into two groups based on endoscopic finings as EGC or AGC [18].

### Stomach cancer-specific CT protocol

Preoperative staging was conducted based on CT scans taken in accordance with a stomach cancer-specific protocol. The equipment was a 16- or 64-detector row CT scan (Sensation 16 or 64; Siemens Medical Solutions, Germany). Each patient fasted for 4 hours before the CT examination. Before the CT scan, patients were injected with 10mg of butylscopolamine bromide (Buscopan; BoehringerIngelheim, Germany) to minimize bowel peristalsis and to induce hypotonia. Images were acquired from the level of the diaphragm to the symphysis pubis with detector collimations of 16×0.75mm or 64×0.6mm. Other scanning parameters were as follows: 160 mAs; 120 kVp; table speed of 24 mm per rotation; and gantry rotation time of 0.5 seconds. For gastric distention, we initially used water as an oral contrast agent (800-1000ml). Beginning in January 2007, we used two packs of effervescent granules for gastric distention. CT scans were obtained with the patient in the supine position irrelevant to the ingested oral contrast. All patients received 120-150ml of contrast medium intravenously using an automatic injector at a rate of 3-4ml/s. Images were acquired in the arterial and portal phases. Axial and coronal images were reconstructed with a 3-mm thickness section and a 3-mm interval.

### Preoperative CT evaluation

The CT scans were evaluated by radiologists specialized in upper gastrointestinal imaging. Mural invasion of gastric cancer into the gastric wall was classified according to previously published criteria [19-22]. A cT1 lesion was defined as non-visualization of an abnormal mural lesion or mucosal thickening with enhancement (cT1a) or visualization of a low attenuation stripe at the base of the lesion, corresponding to the submucosal layer (cT1b). A cT2-3lesion was defined as a thickening of the gastric wall with a loss or disruption of a low attenuating stripe and a clear and smooth outer gastric surface around the lesion. Since CT criteria for cT3 (subserosal layer) lesions have not been established and the serosal layer of the gastric wall is not visible on CT images, differentiation of cT2 (proper muscle layer) from cT3 lesions on CT images is nearly impossible. Thus, we defined cT2 and cT3 lesions as cT2-3. All cT4a lesions demonstrated nodular or irregular outer borders of a thickened gastric wall or perigastric fat infiltration, and all cT4b lesions showed the changes described for cT4a lesions in addition to extension into adjacent organs. Positive lymph nodes were identified on the basis of a size larger than 8 mm along the short axis [2023]. According to this criterion, nodal status on CT scans was classified as follows: cN-, no significant lymph node enlargement, or cN+, lymph node enlargement.

### Pathologic staging

Pathologic evaluation of depth of invasion was performed with resected surgical specimens based on the 7th edition of the International Union Against Cancer Classification (AJCC) as follows: pT1, tumor invasion of the mucosa and/or submucosa; pT2, tumor invasion of the muscularis propria; pT3, tumor invasion of the subserosa; pT4a, tumor penetration of the serosa; and pT4b, tumor invasion of adjacent structures [22, 24]. Lymph nodes were categorized as pN-, no lymph node metastasis, or pN+, more than one lymph node metastasis.

### Grouping of patients

**Figure 2:**
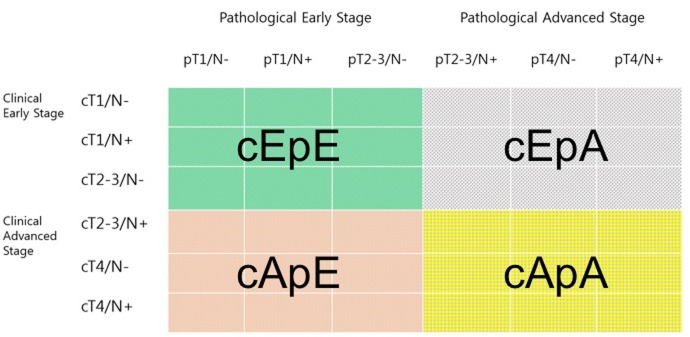
Classification of patients to establish proper selection criteria for treatment modalities The clinical early and pathological early stage (cEpE) group (green color), the clinical early and pathological advanced stage (cEpA) group (gray color), the clinical advanced and pathological early stage (cApE) group (brown color), and the clinical advanced and pathological advanced stage (cApA) group (yellow color).

Although treatment modalities were decided by clinical staging, the appropriateness of the treatment decisions was assessed according to pathologic stage. To establish proper selection criteria for treatment modalities, we grouped patients into one of four groups (Figure 2): (1) the clinical early and pathological early stage (cEpE)group, including clinical stages cT1/N-, cT1/N+, or cT2-3/N- and pathological stages pT1/N-, pT1/N+, or pT2-3/N-; (2) the clinical early and pathological advanced stage (cEpA) group, including clinical stages cT1/N-, cT1/N+, or cT2-3/N- and pathological stages pT2-3/N+, pT4/N-, or pT4/N+; (3) the clinical advanced and pathological early stage (cApE) group, including clinical stages cT2-3/N+, cT4/N-, or cT4/N+ and pathological stages pT1/N-, pT1/N+, or pT2-3/N-; and (4) the clinical advanced and pathological advanced stage (cApA) group, including clinical stages cT2-3/N+, cT4/N-, or cT4/N+ and pathological stages pT2-3/N+, pT4/N-, or pT4/N+. In general, patients in the cEpE and cEpA groups underwent surgery first. Meanwhile, patients in the cApE and cApA groups underwent either surgery or neoadjuvant/perioperative treatment. Between the cApE and cApA groups, patients in the cApA group were more likely to receive more appropriate treatment with accurate clinical staging, which we considered to reflect proper patient stratification. However, cApE patients may have possibly received inappropriate treatment, since the treatment modality was selected according to inaccurate preoperative clinical staging (i.e., improper patient stratification).

### Definition of overestimation and underestimation

Overestimation of the depth of invasion (T) was defined as a case in which the tumor depth on preoperative evaluation by CT scan was deeper than that on final pathologic evaluation. If the tumor depth on preoperative evaluation by CT scan was shallower than that on final pathologic evaluation, T was regarded as being underestimated. Similarly, overestimation of nodal involvement (N) was defined as preoperative CT scans showing significant lymph nodes, despite the absence of an involved lymph node on final pathologic evaluation. Underestimation of N was defined when node-negative gastric cancer on CT scans exhibited the presence of metastatic lymph nodes in the resected specimen. For overall staging, overestimation and underestimation were defined as preoperative clinical stage that was higher or lower than the pathological stage, respectively.

### Statistical analysis

For statistical analysis, SPSS software (version 18.0; SPSS Inc., Chicago, IL, USA) was used. Categorical variables were analyzed using the chi-square test, and continuous data were analyzed using the Mann-Whitney test. For multivariate analysis, logistic regression analysis was used. All values of p less than 0.05 wereconsidered to demonstrate statistical significance.

We assessed reliability by weighted *k* [25]. Weighted *k* was computed to quantify agreement between the CT findings and final pathology. For weighted *k*, full weight was given to perfect agreement; half weight was given to disagreement by one grade; and zero weight was given to other disagreements. The degrees of agreement were categorized as follows: values of 0.00-0.20, poor agreement; values of 0.21-0.40, fair agreement; values of 0.41-0.60, moderate agreement; values of 0.61-0.80, good agreement; and values of 0.81-1.00, excellent agreement [2326].

## Abbreviations

CT: computed tomography
pT: pathological T staging
cT: clinical T staging
pN: pathological N staging
cN: clinical N staging
pT/N: overall pathological staging
cT/N: overall clinical staging
EGC: early gastric cancer
AGC: advanced gastric cancer
cEpE: clinical early and pathological early stage
cEpA: the clinical early and pathological advanced stage
cApE: the clinical advanced and pathological early stage
cApA: the clinical advanced and pathological advanced stage
